# A New Approach to Predicting Cryptocurrency Returns Based on the Gold Prices with Support Vector Machines during the COVID-19 Pandemic Using Sensor-Related Data

**DOI:** 10.3390/s21186319

**Published:** 2021-09-21

**Authors:** Esam Mahdi, Víctor Leiva, Saed Mara’Beh, Carlos Martin-Barreiro

**Affiliations:** 1 Department of Mathematics, Statistics and Physics, Qatar University, Doha 2713, Qatar; smarabeh@qu.edu.qa; 2 School of Industrial Engineering, Pontificia Universidad Católica de Valparaíso, Valparaíso 2362807, Chile; victor.leiva@pucv.cl; 3 Faculty of Natural Sciences and Mathematics, Universidad Politécnica ESPOL, Guayaquil 090902, Ecuador; cmmartin@espol.edu.ec; 4 Faculty of Engineering, Universidad Espíritu Santo, Samborondón 0901952, Ecuador

**Keywords:** artificial intelligence, data science, digital currency, gold, SARS-CoV-2, machine learning, sensing and data extraction

## Abstract

In a real-world situation produced under COVID-19 scenarios, predicting cryptocurrency returns accurately can be challenging. Such a prediction may be helpful to the daily economic and financial market. Unlike forecasting the cryptocurrency returns, we propose a new approach to predict whether the return classification would be in the first, second, third quartile, or any quantile of the gold price the next day. In this paper, we employ the support vector machine (SVM) algorithm for exploring the predictability of financial returns for the six major digital currencies selected from the list of top ten cryptocurrencies based on data collected through sensors. These currencies are Binance Coin, Bitcoin, Cardano, Dogecoin, Ethereum, and Ripple. Our study considers the pre-COVID-19 and ongoing COVID-19 periods. An algorithm that allows updated data analysis, based on the use of a sensor in the database, is also proposed. The results show strong evidence that the SVM is a robust technique for devising profitable trading strategies and can provide accurate results before and during the current pandemic. Our findings may be helpful for different stakeholders in understanding the cryptocurrency dynamics and in making better investment decisions, especially under adverse conditions and during times of uncertain environments such as in the COVID-19 pandemic.

## 1. Introduction

In the last two years, the impact of the 2019 Coronavirus Disease (COVID-19) on the trading strategies between different types of cryptocurrencies, gold, exchange market, portfolio diversification, and macroeconomic policy has attracted a great deal of interest in the financial market [1]. The unexpected recent exponential increase in cryptocurrency prices has exceeded the investors’ imaginations. However, this does not indicate that cryptocurrencies are a substitute for gold [2]. For more than 2000 years, gold has been known as a high-quality liquid asset that has served as a hedge and safe haven for exchanging commodities. Several robust statistical models have been proposed to explore the predictability of the gold price [2,3].

Cryptocurrencies are digital currencies that belong to a decentralized system still classified in the nascent asset class [4]. Their prices are very sensitive to good and bad news with significant instability leading to high volatility and making the predictions hard and challenging, especially under adverse market conditions [2,4]. Thus, investments in cryptocurrencies are, often, equated to investments in gold, where cryptocurrencies may benefit from higher allocations to gold and vice-versa [2].

The emergence of blockchain [5] and decentralized cryptocurrencies has been a catalyzed innovation in the financial market. Before the global outbreak of COVID-19, research focused on using the Granger-causality and quantile-on-quantile regression (QQR) [6,7] to model the relationship between price change of crude oil, gold, denominated currencies, and other financial markets, on the one hand, and price change of top-ranked cryptocurrencies, on the other hand [8,9,10,11,12].

In the wake of the COVID-19 pandemic, several studies were conducted to explore the influence of the pandemic intensity on the gold and cryptocurrency market. For example, the asymmetric impacts of COVID-19 on the multifractality of oil and gold prices (and vice-versa) based on upward and downward trends were examined in [13]. They showed that the gold and oil markets during the pre-COVID-19 period were more inefficient during downward (upward) trends, but this has changed during the outbreak period [14].

A type of multivariate GARCH model, named VAR-AGARCH, was used in [15] to examine the return and volatility spillover of Bitcoin (BTC), Ethereum (ETH), and Litecoin (LTC) cryptocurrencies before and during the COVID-19 period. The authors [15] did not compare the performance of the VAR-AGARCH model with modern techniques as machine learning algorithms [16]. The QQR was employed in [17] to explore the asymmetric impact between the changing intensity in the quantiles of COVID-19 and the quantiles of the daily returns of the top-ranked cryptocurrencies. Their numerical results are based on data collected over the first six months of the outbreak of the pandemic. By utilizing these results, contrary to what was indicated in [18], the QQR suggested that BTC, Cardano (ADA), and ETH performed better in comparison with other currencies.

The GARCH-MIDAS model adopted in [4] examined the impact of news, during COVID-19, on the predictability of return volatility of cryptocurrencies. They showed that the pandemic increases the volatility of digital currencies compared with the period before the outbreak of COVID-19. A significant causal relationship was shown in [19], in quantiles, between the US dollar and the top five cryptocurrencies during the pandemic, which had a large effect on the digital currencies’ relationship.

The impact of the COVID-19 lockdowns on the BTC trading volume was analyzed in [20] by using Apple mobility volume trends and autoregressive-moving-average models [21]. The authors [20] reported that the investors traded more BTCs during the days with low mobility associated with lockdown mandates, even after controlling the stocks and gold prices.

In many applications, the support vector machine (SVM) technique [22], a machine learning tool, has shown a superior forecasting performance in linear and non-linear models, especially when the data have outliers, compared with most evolutionary algorithms [23]. SVM can be classified into two algorithms: support vector classification (SVC) and support vector regression (SVR) [24]. Both algorithms are computationally intensive and require a large amount of memory, but with the age of big data [25] and quantum computing, this can be affordable. Interest in these algorithms has increased in the cryptocurrency market and it deserves to continue. The discriminant analysis (DA) and SVM algorithms were utilized in [26] to model the subsurface gold mineralization in the northwestern part of Iran. They compared the performance of four classification functions of DA and SVM methods and showed that the SVM algorithm worked better than the DA method.

SVM algorithms were applied in [27] to investigate the profitability of the cryptocurrency market and explore how high they would be. The authors showed that the SVM technique could outperform the benchmark strategies in terms of return-risk relation. They showed that the SVR algorithm provides better results in comparison to ARIMA models. An SVM algorithm based on particle swarm optimization (SVM-PSO) algorithm [28] was proposed in [29] to forecast the future price of cryptocurrency. They reported that the performance of the SVM-PSO technique is better than the SVM algorithm. The ARIMA and SVR models were employed in [3] to predict the daily price of gold, whereas in [30], the SVC model was utilized to predict whether the price of three cryptocurrencies (BTC, ETH, and LTC) would increase or decrease in the next day.

Note that traditional econometric and time-series models, such as the ARMA and GARCH models, might not provide helpful results in predicting cryptocurrency returns due to the impact of risk and uncertainty on return volatility. Although several SVM techniques have already been used in numerous applications, very few have been done in the cryptocurrency market. To the best of our knowledge, the SVM has never been employed to predict the daily classification of cryptocurrency returns according to the quantiles of the gold price the next day.

Our objective is to propose a new approach for predicting cryptocurrency returns by using the SVC technique. Specifically, instead of forecasting the movement of the cryptocurrency return indicating whether it would go up or down, we forecast the type of this return reporting whether it would be in the first, second, third quartile, or any quantile of the gold price the next day. Therefore, our investigation fills this gap by proposing a new methodology for predicting the daily classification of cryptocurrency returns according to the quantiles of the gold price the next day. We classify the returns of the six major digital currencies (ADA, Binance Coin –BNB–, BTC, Dogecoin –DOGE–, ETH, and Ripple –XRP–) selected from the list of top ten cryptocurrencies into four categories according to the quartiles of the gold price values. In the current COVID-19 contingency scenario, data of cryptocurrency returns and COVID-19 are often collected through sensors [31,32,33].

The rest of this article is organized as follows. In Section 2, we propose an algorithm that uses a sensor in the database to predict the return of cryptocurrencies considering COVID-19 data and the price of gold. Section 3 defines the methodology based on the SVM models employed in this study. In Section 4, we present the data and results of our investigation, whereas in Section 5 our conclusions are reported.

## 2. Sensing Data of Cryptocurrencies, Gold, and COVID-19

To perform the empirical analysis proposed in this work (see Section 4), a software has been implemented with a local database that uses the SVM algorithm considering two aspects. In addition, we design an algorithm that allows us to sense changes in the data and show updated results. The database management system permits the configuration of a digital sensor. This sensor can detect if new data are entered into the database, if the data are modified, or if data are deleted.

In this work, a sensor that detects the presence of new data is utilized. As a response to new data, a trigger is implemented, which enables instructions to be registered and executed when the new data arrive. This sensor permits the software that performs the calculations to be notified that new records were entered in the database and therefore that the computations must be updated.

In summary, the local database is updated daily with external data (websites). A sensor in the local database sends a signal to the developed software to perform the calculations considering the new data. The software receives the signal and executes the corresponding processing. Lastly, the software delivers the results.

Next, we describe Algorithm 1 that senses changes in the data and shows updated results. For the sake of better understanding, we relate some steps of this algorithm to our case study in Section 4.
**Algorithm 1** Approach to predict the daily classification of cryptocurrencies returns relating them to gold price and COVID-19 data using sensors.1:Update table of deaths and infected cased due to COVID-19 with the data extracted from the website https://github.com/CSSEGISandData/COVID-19 (accessed on 29 August 2021) and use this table for constructing the data warehouse.2:Update table of gold prices with the data extracted from the website https://www.gold.org/goldhub/data/gold-prices (accessed on 29 August 2021) and employ this table for building the data warehouse.3:Update table of cryptocurrency returns taking the daily closing price and the market capitalization of the six studied digital currencies with the data extracted from the website https://coinmarketcap.com (accessed on 29 August 2021) and utilize this table for creating the data warehouse.4:Send a warning to the software that performs a data analysis when new records are detected by means of the trigger. Note that the database has a digital sensor with the ability to detect when new data are entered.5:Communicate the trigger (in the database) with the software through a middleware so that the data analysis may be performed by executing the following steps:5.1 Plot Figures 2–4, and construct Table 1.5.2 Use the probability density function to state the type of skewness and kurtosis of the data empirical distribution and describe the shape of their distribution.5.3 Employ the Jarque-Bera (JB) test and elaborate Table 2.5.4 Execute the SVM algorithm considering the one-to-one method (see Section 3), that is, separate the observations into training and testing data sets, and select the best model based on the indicators defined in (11).5.5 Apply the SVM algorithm considering the one-to-rest method (see Section 3) and build a dynamic model based on the available data.5.6 Construct Figures 5–7.6:End the algorithm reporting the results of the updated data analysis in the graphical user interface of the software.

## 3. Methodology

SVM with binary classification is a powerful learning algorithm that aims to differentiate between two classes by finding an optimal hyperplane that maximizes the separation between the two-dimensional space points [23].

An algorithm that maximizes the margin between the training patterns and the decision boundary is a helpful tool that can be applied to a wide variety of classifiers. In this tool, the number of parameters must be fit automatically to match the complexity of the problem, with the solution being formulated as a linear combination of support patterns, which are a subset of training patterns that are closest to the decision boundary.

Next, we provide a background of the method used in the present investigation. For detailed mathematical derivations of the results presented in this section, we refer the readers to [34].

For *m*-dimensional space points, with 2<m∈Z, we cannot utilize the binary SVM to obtain the separation hyperplane (decision surface) directly. To overcome this issue, the multi-classification problem breaks down into multiple binary classification problems. Then, the binary SVM algorithm is applied to these binary classes. This may be done in a one-to-one or one-to-rest manner explained as follows:

- One-to-one method: Here, we break down the *m* classes into m(m−1)/2 mutual binary classes, where a binary SVM is employed to differentiate between every two binary classes. Each binary SVM finds a hyperplane that separates between every two classes, neglecting the data points of the other classes. For example, consider m=4, say, with classes 1,2,3, and 4. In this case, we have six different SVMs applied to the binary classes {1,2},{1,3},{1,4},{2,3},{2,4}, and {3,4}.
- One-to-rest method: Here, the classifier can use *m* binary SVMs by obtaining an optimal hyperplane that separates between a class and all others at once. For example, consider m=3, say, with classes 1,2, and 3. In this case, we have three different SVMs applied to the binary classes {1,not 1}, {2,not 2}, and {3,not 3}.

The data points classification depends on the type of the kernel function to be employed, which aims to reduce the computational time and effort by transforming a non-linear decision surface to a linear equation in a higher number of dimension spaces. For any two vectors u and v, the most popular kernels, utilized with SVM algorithms, are linear, polynomial, radial basis, and sigmoid functions defined as:

- Linear function:
  (1) $$K\left(u,v\right)=u^{⊤}v,$$
  where *K* is the kernel function and ${}^{⊤}$ denotes the transpose of a vector.
- Polynomial function:
  (2) $$K\left(u,v\right)={\left(\gamma u^{⊤}v+1\right)}^{d},$$
  where γ>0 and d>0 are the parameters of scale and degree, respectively.
- Radial basis function:
  (3) $$K\left(u,v\right)=\exp(-\gamma ||u-v|{|}^{2}),$$
  where γ>0 is the inverse influence radius of points selected as support vectors.
- Sigmoid function:
  (4) $$K\left(u,v\right)=\tanh\left(\gamma u^{⊤}v+1\right),$$
  where γ>0 is a tuning meta-parameter that defines how far the influence of a training point can reach.

Let $\left(x_{1},y_{1}\right),\cdots ,\left(x_{n},y_{n}\right)$ be the training data set, where each pair belongs to one of two classes that we name A or B, according to

$$y_{k}=\left\{\begin{array}{cc} 1, & \mathrm{if}\;x_{k}\in \mathrm{class}\;A;\\ -1, & \mathrm{if}\;x_{k}\in \mathrm{class}\;B. \end{array}\right.$$

**Figure 1:**
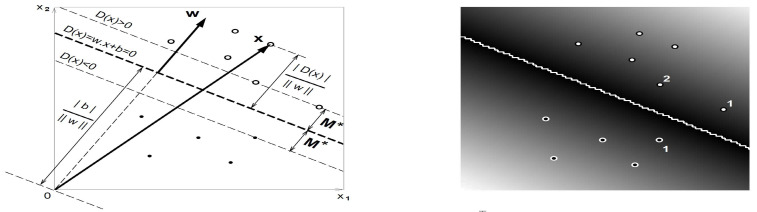
Plot of the maximum margin linear decision function $D\left(x\right)=w^{⊤}x+b\;\left(\varphi \left(x\right)=x\right)$ (**left**) and an image of it (**right**). The gray levels encode |D(x)|, solid black corresponds to D(x)=0, and the numbers indicate the supporting vectors (Source: [34]).

Each $x_{k}$, with k=1,⋯,n, is a *p*-dimensional vector of real numbers. From the training data set, the maximum margin hyperplane algorithm is used to estimate the parameters of the decision function D(x). Hence, the classification of unknown patterns is predicted based on the following criteria: (i) x∈classAifD(x)>0; or (ii) x∈classB, otherwise. In the direct space, D(x) has the form $D\left(x\right)=w^{⊤}\varphi \left(x\right)+b,$ where φ(x) is a predefined function, which separates the hyperplane in *n*-dimensional spaces, whereas $w={\left(w_{1},\cdots ,w_{n}\right)}^{⊤}$ and b are vectors of weights and noise coefficients, respectively, with both w and b being adjustable parameters computed along with minimizing the empirical risk. The distance between the pattern x and the separated hyperplane is D(x)/||w|| (see Figure 1). Note that the axes in this figure (left) are labeled as x${}_{1}$ and x${}_{2}$. Nonetheless, the figure on the right-hand side is the same as that on the left-hand side but as an image (photography).

The maximum margin hyperplane algorithm aims to find the vector w that maximizes the margin *M* between the training set and class boundary, which is formulated as

(5) $$\begin{array}{ccc} M^{☆} & = & \max_{w,||w||=1}M\\ \mathrm{subject}\mathrm{to}\;y_{k}D\left(x\right) & \geq & M,\;k=1,\cdots ,p, \end{array}$$

where the bound $M^{☆}$ for supporting patterns attains to satisfy the condition $M^{☆}=\min_{k}\;y_{k}D\left(x_{k}\right)$. The decision function, D(x) namely, with maximum margin $M^{☆}$, is shown in Figure 1. Therefore, the hyperplane with maximum margin can be obtained by solving the minimax problem established as $\max_{w,||w||=1}\min_{k,b}\;y_{k}D\left(x_{k}\right).$ Usually, the product of the norm of w and the margin *M* is scaled to be fixed, that is, M||w||=1, to avoid the problem of getting an infinite number of possible solutions. Therefore, maximizing *M* as in (5) is equivalent to minimizing ||w||. Thus, finding the hyperplane with maximum margin ($M^{☆}=1/\left|\right|w^{☆}\left|\right|$) reduces to solve the quadratic form given by

(6) $$\begin{array}{ccc} \min_{w,b}{\;||w||}^{2},\\ \mathrm{subject}\mathrm{to}\;y_{k}D\left(x_{k}\right) & \geq & 1,\;k=1,\cdots ,p. \end{array}$$

Often, we may tolerate some cost errors ξ≥0 by allowing for some observations to violate the margin. The cost *C* is defined by the number of observations beyond the margin. In this case, instead of minimizing the form stated in (6), we need to minimize

(7) $$\begin{array}{ccc} \min_{w,b}\;\frac{1}{2}{\left||w|\right|}^{2} & + & C\sum_{k=1}^{p}\xi_{k},\\ \mathrm{subject}\mathrm{to}\;y_{k}D\left(x_{k}\right) & \geq & 1-\xi_{k},\;k=1,\cdots ,p, \end{array}$$

where $\xi_{k}\geq 0$ and $\sum_{k=1}^{p}\xi_{k}\leq C$. However, although the problem stated in (7) can be solved by numerical methods in direct space, these methods are impractical for high dimensionality of the dual space φ. In this dual space, the decision function is defined as

(8) $$D\left(x\right)=\sum_{k=1}^{p}\alpha_{k}K\left(x_{k},x\right)+b,$$

where $0\leq \alpha_{k}\leq c$ are adjustable parameters computed along with minimizing the empirical risk, *C* is the cost given by the number of observations outside the margin, and *K* is a predefined kernel function. The values of the kernel function stated in (8) may be calculated by the inner product of $\varphi (x_{i})$ and $\varphi (x_{j})$, that is, $K\left(x_{i},x_{j}\right)=\varphi \left(x_{i}\right)\circ \varphi \left(x_{j}\right)$, where the operator ∘ denotes the inner product of two vectors. We aforementioned the most popular kernels in the expressions defined in (1) to (4). The problem stated in (7) can be transformed into a dual space using a Lagrangian algorithm [31] by means of the representation formulated as

(9) $$L\left(w,b,\alpha \right)=\frac{1}{2}{\left||w|\right|}^{2}-\sum_{k=1}^{p}\alpha_{k}\left(y_{k}D\left(x_{k}\right)-1\right),$$

under the condition that the Lagrangian multipliers $\alpha_{k}\geq 1$, for k=1,⋯,p [35].

Note that the tolerance errors are again introduced, defining ξ as the value outside the error margins and $\xi^{☆}$ to limit the value to the dependent variable. Thus, the Lagrangian dual of the problem stated in (9) may be solved by minimizing the expression given by

(10) $$\frac{1}{2}{\left||w|\right|}^{2}+c\sum_{k=1}^{p}\left(\xi_{k}-\xi_{k}^{☆}\right),$$

under the conditions $\left(w^{⊤}x_{k}+b\right)-y_{k}\leq 1+\xi_{k}$ and $y_{k}-\left(w^{⊤}x_{k}+b\right)\leq 1+\xi_{k}^{☆}$.

## 4. Empirical Analysis with Real-World Data

To conduct our study with real-world data, we employ the SVC algorithm proposed in [23]. We use the R software with the e1071 [36] and ggplot2 [37] packages to carry out the data analysis [38]. As mentioned, we perform our analysis using three type of data: (i) daily confirmed COVID-19 infections and deaths; (ii) daily gold price –in US dollars–; and (iii) daily closing price of major cryptocurrencies—in US dollar—.

**Figure 2:**
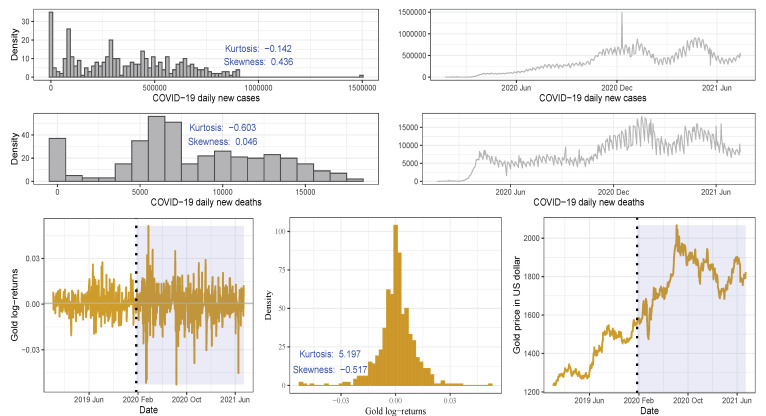
Probability density function and trend of daily (from 23 January 2020 to 01 July 2021) confirmed COVID-19 infections and deaths (**upper and middle**) and daily (from 04 December 2018 to 14 July 2021) returns (**lower left**), probability density function (**lower middle**), and prices (**lower right**) of the gold. The vertical dotted line represents the threshold between the pre-COVID-19 period (**left**) and during the COVID-19 period (**right**) so that the shaded zone represents the period after the pandemic begins to visualize its impact on the gold price.

**Figure 3:**
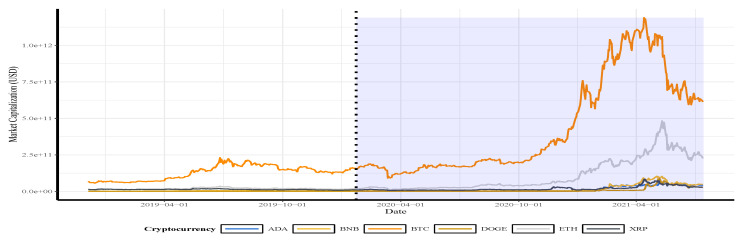
The market capitalization of the indicated cryptocurrencies. The vertical dotted line represents the threshold between the pre-COVID-19 period (**left**) and during the COVID-19 period (**right**) so that the shaded zone represents the period after the pandemic’s beginning to visualize its impact on the cryptocurrency return.

**Figure 4:**
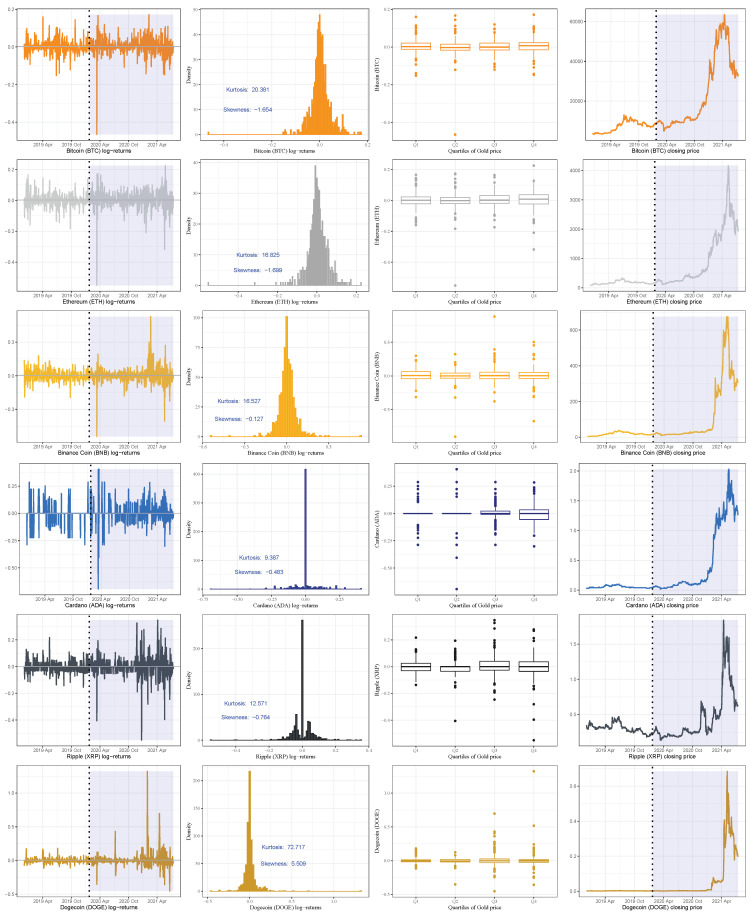
Cryptocurrency daily returns and box-plots of these returns based on the quartiles of the gold prices. The vertical dotted line represents the threshold between the pre-COVID-19 period (**left**) and during the COVID-19 period (**right**) so that the shaded zone represents the period after the pandemic’s beginning to visualize its impact on the cryptocurrency return.

**Figure 5:**
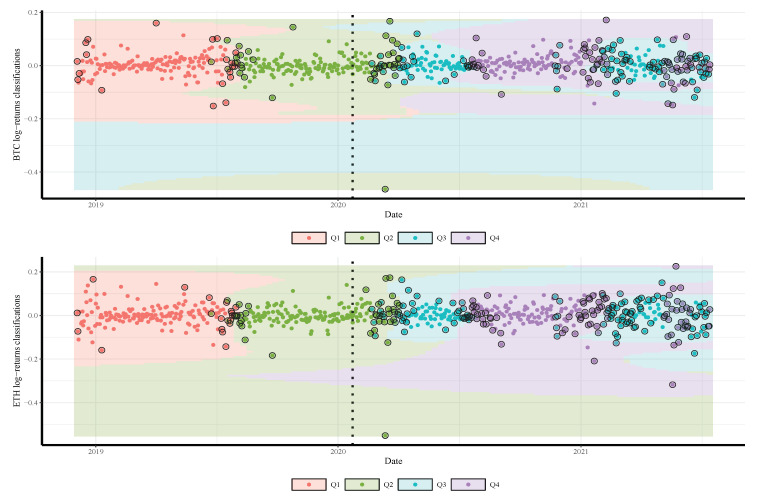
Classifications of BTC (**top**) and ETH (**bottom**) daily returns based on quartiles of daily gold prices predicted by the SVM model. The vertical dotted line represents the threshold between the pre-COVID-19 period (**left**) and ongoing COVID-19 period (**right**) to visualize its impact on the cryptocurrency return.

**Figure 6:**
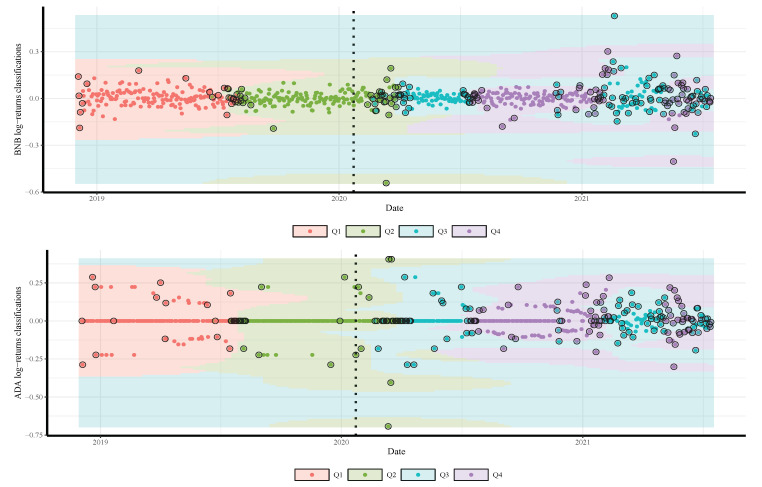
Classifications of BNB (**top**) and ADA (**bottom**) daily returns based on quartiles of daily gold prices predicted by SVM model. The vertical dotted line represents the threshold between the pre-COVID-19 period (**left**) and ongoing COVID-19 period (**right**) to visualize its impact on the cryptocurrency return.

**Figure 7:**
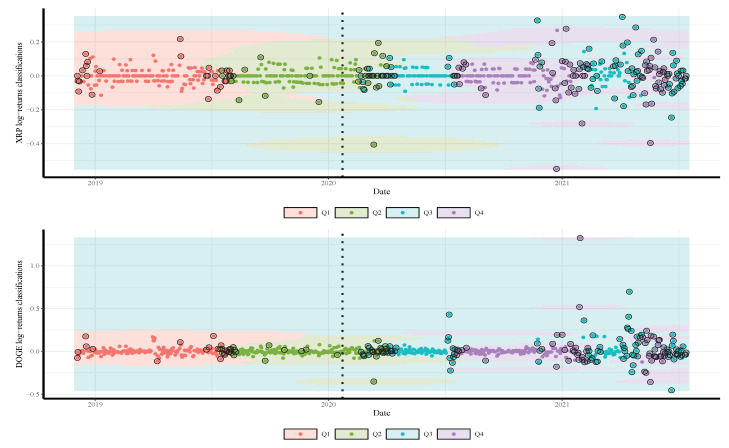
Classifications of XRP (**top**) and DOGE (**bottom**) daily returns based on quartiles of daily gold prices predicted by SVM model. The vertical dotted line represents the threshold between the pre-COVID-19 period (**left**) and ongoing COVID-19 period (**right**) to visualize its impact on the cryptocurrency return.

- Daily confirmed COVID-19 infections and deaths:
  First, we collect the daily confirmed COVID-19 infections and deaths from the online repository https://github.com/CSSEGISandData/COVID-19 (accessed on 29 August 2021). This web-hosting has been operated by the Johns Hopkins University Center for Systems Science and Engineering from 23 January 2020 until the present, and it is updated twice daily. We consider the period from 23 January 2020 to 14 July 2021. This period essentially accounts for all available data officially published [39] up to the date where we started this research.
- Daily gold price in US dollars:
  Second, to compare the impact of the pre-COVID-19 period with the COVID-19 period on the classification of cryptocurrency returns according to the quantile values of the gold price (in US dollars), we collected the gold price data from the website https://www.gold.org/goldhub/data/gold-prices (accessed on 29 August 2021) over the period 04 December 2018 to 14 July 2021. In this article, the word return is used synonymously with log returns.
  The four graphs in the upper and middle of Figure 2 present the distribution and trend of the daily infections and deaths due to COVID-19. The graphs show that, for more than 100 days, the number of daily deaths ranged from 5000 to 7500 cases. The plots in Figure 2 show that the daily confirmed COVID-19 infections and deaths and the daily gold prices followed a similar trend during the period of the ongoing COVID-19 pandemic. The gold prices report high volatility clustering in the pandemic compared with the pre-COVID-19 period. For the entire period of study, the probability density function of the gold prices is slightly skewed to the left with a high excess kurtosis.
  *Remark.* Note that the shaded region in Figure 2, Figure 3 and Figure 4 describes the period after the beginning of the pandemic for better visualization of the impact of COVID-19 on the gold price/cryptocurrency return. Also, in these figures and in Figure 5, Figure 6 and Figure 7, the vertical dotted line represents the threshold between the pre-COVID-19 period and ongoing COVID-19 period.
- Daily closing price of major cryptocurrencies in US dollars:
  Third, we collect the daily closing price and market capitalization for some major cryptocurrencies available from the online source https://coinmarketcap.com (accessed on 29 August 2021). For the current research, as mentioned, we study the six cryptocurrency assets, that is, ADA, BNB, BTC, DOGE, ETH, and XRP, selected from the top 10 ranked currencies.
  Table 1 provides the main characteristics of the top-ranked cryptocurrencies and Figure 3 presents the market capitalization of used currencies over the study period. Again, the vertical dotted lines represent the threshold between the two periods of time (before and during the pandemic). The trends show an exponential increase in the market capitalization of BTC and ETH and less increase in the other currencies during the period of the outbreak of the pandemic.

Almost all currencies followed a similar trend during the period of study. The graphical plots also indicate that the market capitalization substantially plummeted, from mid-May 2021 until the end of the study period, when Tesla (https://www.bbc.com/news/business-57096305, accessed on 29 August 2021) announced that it would not accept or sell the cryptocurrencies due to pollution concerns. Such bad news often impacts the cryptocurrency prices leading to a substantial increase in the volatility of the returns. Thus, the traditional time-series models might not provide accurate cryptocurrency returns forecasting results, especially during adverse market conditions. Then, the SVM model might be a good option to achieve forecasting accuracy as we will show next.

We split the gold price into four classes according to their quartiles and labeled the values as $Q_{1},Q_{2},Q_{3}$, and $Q_{4}$. Note that, although our approach can work for all quantiles (from 0 to 100), we consider only four quartiles for simplicity’s sake. Readers may use the supplementary source code for any subset of gold-price quantiles. We combine the quartiles of the gold prices one day in advance with the daily log-returns of the employed currencies and the COVID-19 cases and deaths. The resulting date corresponds to a time series comprised of 682 observations from 04 December 2018 to 22 January 2020 (before COVID-19 with 297 observations) and from 23 January 2020 to 14 July 2021 (during COVID-19 with 385 observations).

Figure 4a,b illustrate the daily returns, distribution, box-plot, and prices of the studied currencies. The box-plots illustrate that the cryptocurrency returns have many outliers in both periods and the volatility of the returns during the COVID-19 period is considerably higher than in the pre-COVID-19 pandemic period.

Table 2 summarizes the statistics of the daily gold prices and returns of the studied currencies. The averages of the gold price and all cryptocurrencies returns have substantially increased over time, especially during the ongoing COVID-19 pandemic. The *p*-values based on the JB test strongly reject the hypothesis that the returns are normally distributed, which is coherent with the graphical analysis. During the entire period of the study, gold and currencies have a significant excess kurtosis with negative skewness (except the probability density function of the DOGE returns which has a positive skewness), as noted in Figure 2, Figure 3 and Figure 4.

To examine for a bi-directional causal relationship between the daily number of newly infected cases (deaths) of COVID-19 and the daily gold price (cryptocurrencies returns), we apply the Granger causality test at a lag equal to three. The results are reported in Table 3, which reports that no Granger causality exists. In contrast, we find a significant casualty between the quartiles of the gold price and almost all of the cryptocurrencies returns the next day. According to the findings, contrary to the BNB and DOGE, the returns of the ADA, BTC, and ETH can affect the quartile value of the gold price, but the opposite is not true. In addition, we do not find enough evidence for a causal relationship between the XRP returns and the quartiles of the gold prices.

The results in Table 2 and Figure 2, Figure 3 and Figure 4 also show a significant increase in the volatility of the returns of gold and currencies after the outbreak of the COVID-19, compared with the pre-pandemic period. Note that, in these figures, as mentioned, the vertical dotted line represents a threshold between the pre-COVID-19 period and during the pandemic. In addition, the circled points are the support vectors, and misclassification appears when the color of the support vectors does not match the color of the decision boundary. Thus, the traditional econometric and time-series models might not provide helpful results in such cases. Hence, due to its superior forecasting performance, we implement the SVM in our analysis.

The SVM optimization strategy is usually done in two stages. First, we separate the observations into training and testing data sets. A rule of thumb indicates to consider the first 75% contiguous days as a training set to fit the model and the remaining 25% days as a testing set, which is used to validate the prediction accuracy of this model [40]. The best model is selected based on minimizing the mean absolute percentage error (MAPE), root mean square error (RMSE), or normalized root mean square error (NRMSE) defined as

(11) $$\begin{array}{ccc} \mathrm{MAPE} & = & \frac{1}{N}\sum_{i=1}^{N}\left|\frac{y_{i}-{\hat{y}}_{i}}{y_{i}}\right|\times 100\%,\\ \mathrm{RMSE} & = & \sqrt{\frac{1}{N}\sum_{i=1}^{N}{\left(y_{i}-{\hat{y}}_{i}\right)}^{2}},\\ \mathrm{NRMSE} & = & \sqrt{\frac{N\sum_{i=1}^{N}{\left(y_{i}-{\hat{y}}_{i}\right)}^{2}}{{\left(\sum_{i=1}^{N}y_{i}\right)}^{2}}}, \end{array}$$

where *N* is the total number of observations, ${\hat{y}}_{i}$ is the predictive value of $y_{i}$, for i=1,⋯,N, and $y_{i}-{\hat{y}}_{i}$ denotes the prediction error in $y_{i}$.

Second, we utilize the SVM optimization strategy as a dynamic model that involves all available data. In [41], it is showed that the dynamic model can achieve more accurate forecasting results compared with the one based on a fixed training set. Therefore, in this article, we adopt the dynamic SVM algorithm.

The optimized estimation of the coefficient *c* stated in (10) and the parameters γ,d defined in (2) and (3), respectively, may be obtained based on the *k*-fold cross validation method, where the index expressed in (11) has the mimimum value. In this article, we consider a 10-fold cross validation and a set of tuning parameters c∈{0.001,0.01,1,2,2,4,8,16,32,64,128,256},d∈{1,2,3}, and γ∈{0.3,0.4,0.5}. The estimated parameters of the linear, polynomial, and radial kernel models for the entire sample are shown in Table 4. Applying the optimized parameters in Table 4, the SVM models with each kernel are run on a daily series and the forecasting performance accuracy (in %) for each currency is calculated. The results are indicative of the SVM’s superior predictive power when using a radial kernel (between 89.4% and 91.8% performance accuracy) compared with linear (between 71.8% and 74.5% performance accuracy) and polynomial (between 74.8% and 79.3% performance accuracy) kernels.

To illustrate the SVM classification prediction capabilities, using the radial classifier, during both periods, pre- and during the COVID-19 outbreak, Figure 5, Figure 6 and Figure 7 show the decision rules for a different set of patterns in the hyperplane. The four classes of gold price quartiles are represented by dotted points with four different colors. In the figures, we indicate the support vectors (patterns) with dotted points and double circles, and the errors (misclassifications) with dotted points and double circles but in different colors. Figure 5, Figure 6 and Figure 7 display that the majority of the errors occurred during the COVID-19 period compared with the pre-period of COVID-19, where some of the cryptocurrencies returns from $Q_{3}$ class of the gold price are misclassified as $Q_{4}$. This is justifiable as the uncertainty about the COVID-19 has resulted in significant effects that continue to drive perceptions of risk and volatility in cryptocurrencies and gold prices.

## 5. Conclusions

In this study, we have applied the support vector machine model to daily returns of six major cryptocurrencies: Binance Coin, Bitcoin, Cardano, Dogecoin, Ethereum, and Ripple, before and during the COVID-19 pandemic. The daily returns and volatility spillover substantially increased during the period of the pandemic, due to the high fluctuation in prices in comparison with the pre-pandemic period, making accurate forecasting a complex duty. Instead of using a traditional time series model to predict the return of the cryptocurrencies, we have proposed a new easy approach based on an advanced machine learning algorithm to achieve accurate forecasting results for the classification of the returns.

We have designed an algorithm that allows us to use sensors when submitting requests for data of cryptocurrency returns, gold prices, and COVID-19. After collecting the price series of the gold and a cryptocurrency, one can classify the returns of the cryptocurrency in the previous day based on the quartile of the gold price the next day. Also, we may employ the proposed model to predict the classification of the return, whether it will be in the first, second, third or fourth quartile of the gold price.

We have shown that the SVM model can provide significant accurate forecasting, especially during crises. Overall, our model is not only valuable for predicting the classification of the returns of the aforementioned cryptocurrencies, but it is also helpful to be used for other cryptocurrencies.

Our findings support the claim that the SVM technique provides a robust algorithm for the predictability of cryptocurrencies under bad or good news. We believe that the findings of this research are not only helpful for exploring the relationship between the classification returns of the cryptocurrencies and the quantiles of the gold price, but they are also of great interest to macroeconomic policymakers, portfolio managers, and investors who are actively trading in the cryptocurrency market, especially under adverse market conditions and during times of uncertainty. In summary, our new approach can help to devise trading strategies for cryptocurrencies and help investors and macroeconomic policymakers making better investment decisions.

This article provides avenues for further research in digital currency portfolios by comparing our model with other existing evolutionary machine learning algorithms to achieve more accurate results.

## Figures and Tables

**Table 1:** Characteristics of the top ten cryptocurrencies (14 July 2021). Source: https://coinmarketcap.com (accessed on 29 August 2021).

Rank	Name	Symbol	Price (in US $)	Market Capitalization (in US $)
1	Bitcoin	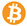 (BTC)	32,822.35	615,635,042,692
2	Ethereum	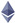 (ETH)	1,994.33	232,727,182,406
3	Tether	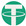 (USDT)	1.00	62,093,784,456
4	Binance Coin	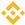 (BNB)	309.41	47,473,117,237
5	Cardano	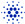 (ADA)	1.26	40,444,089,747
6	Ripple	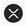 (XRP)	0.62	28,669,334,327
7	USD Coin	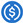 (USDC)	1.00	26,668,074,913
8	Dogecoin	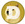 (DOGE)	0.20	25,738,852,158
9	Polkadot	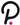 (DOT)	13.67	13,310,832,721
10	Binance USD	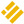 (BUSD)	1.00	11,579,221,385

**Table 2:** Descriptive statistics and preliminary tests for data of gold price, cryptocurrency return, and COVID-19.

Variable	Period	Min	$Q_{1}$	Mean	Median	$Q_{3}$	Max	SD *	JB Test **
Gold	Pre-COVID-19	1235	1292	1391	1400	1490	1573	101.29	31
	COVID-19	1474	1720	1792	1800	1880	2067	113	12
	Both periods	1235	1429	1617	1617	1817	2067	226.07	51
BTC	Pre-COVID-19	−0.1518	−0.0156	0.0018	0.0009	0.0188	0.1600	0.0376	137
	COVID-19	−0.4647	−0.0147	0.0036	0.0033	0.0232	0.1718	0.0477	9767.9
	Both periods	−0.4647	−0.0151	0.0028	0.0018	0.0210	0.1718	0.0436	12196
ETH	Pre-COVID-19	−0.1833	−0.0212	0.0003	−0.0012	0.0183	0.1661	0.0453	96
	COVID-19	−0.5507	−0.0203	0.0043	0.0052	0.0354	0.2256	0.0618	5793
	Both periods	−0.5507	−0.0204	0.0026	0.0016	0.0293	0.2256	0.0552	8429
BNB	Pre-COVID-19	−0.1929	−0.0235	0.0027	0.0005	0.0310	0.1787	0.0489	43
	COVID-19	−0.5428	−0.0216	0.0058	0.0038	0.0327	0.5292	0.0752	4002.3
	Both periods	−0.5428	−0.0232	0.0045	0.002	0.0323	0.5292	0.0650	7818
ADA	Pre-COVID-19	−0.2877	0.0000	−0.0018	0.0000	0.0000	0.2877	0.0777	317
	COVID-19	−0.6931	−0.0082	0.0042	0.0000	0.0217	0.4055	0.0940	1817
	Both periods	−0.6931	0.0000	0.0016	0.0000	0.0000	0.4055	0.0873	2550
XRP	Pre-COVID-19	−0.1542	−0.0317	−0.0018	0.0000	0.0000	0.2171	0.0445	109
	COVID-19	−0.5486	−0.0339	0.0011	0.0000	0.0392	0.3474	0.0814	1657
	Both periods	−0.5486	−0.0328	−0.0002	0.0000	0.0317	0.3474	0.0678	4590
DOGE	Pre-COVID-19	−0.1156	−0.0182	0.0003	−0.0024	0.0133	0.1803	0.0377	452
	COVID-19	−0.4523	−0.0244	0.0091	−0.0006	0.0218	1.3235	0.1163	36,238
	Both periods	−0.4523	−0.0208	0.0053	−0.0017	0.0179	1.3235	0.0909	154,646

* SD stands for standard deviation and ** all JB *p*-values are significant at 1%.

**Table 3:** Granger causality test results for data of gold prices, cryptocurrency return, and COVID-19.

COVID-19 Infected Cases				COVID-19 Death Cases		
**Null Hypothesis**	**Wald**	**p-Value**		**Null Hypothesis**	**Wald**	***p*** **-Value**
COVID-19 /⇒Gold	0.4376	0.7262		COVID-19 /⇒Gold	0.1968	0.8985
COVID-19 /⇒BTC	0.9753	0.4044		COVID-19 /⇒BTC	0.6815	0.5638
COVID-19 /⇒ETH	1.9525	0.1207		COVID-19 /⇒ETH	0.8400	0.4726
COVID-19 /⇒BNB	0.7258	0.5371		COVID-19 /⇒BNB	0.2029	0.8944
COVID-19 /⇒ADA	1.0662	0.3634		COVID-19 /⇒ADA	2.0982	0.1000
COVID-19 /⇒XRP	2.4939	0.0597		COVID-19 /⇒XRP	2.2387	0.0834
COVID-19 /⇒DOGE	0.8942	0.4442		COVID-19 /⇒DOGE	2.2739	0.0796

**Table 4:** Optimal parameters and performance accuracy (in %) for the indicated currency and kernel.

Currency	Linear Kernel		Polynomial Kernel			Radial Kernel		
	Accuracy	*c*	Accuracy	*c*	*d*	Accuracy	*c*	γ
Binance Coin	73.2	64	76.8	512	3	91.5	512	0.4
Bitcoin	74.5	256	77.3	256	3	91.8	512	0.3
Cardano	72.6	256	74.8	512	3	91.1	128	0.4
Dogecoin	71.8	256	76.5	512	3	89.4	16	0.4
Ethereum	73.0	64	79.3	128	3	90.9	128	0.3
Ripple	72.3	128	75.8	512	3	90.8	256	0.3

Where the parameter *c* is used for all kernels, *d* is the degree parameter of the polynomial kernel, and γ is the radial kernel parameter.

## Data Availability

The analyzed data and used codes are available under request.
